# Disposal of unnecessary and expired medicines in households: Practices and factors associated with proper medicine disposal using the extended Theory of Planned Behavior

**DOI:** 10.1016/j.rcsop.2026.100799

**Published:** 2026-05-12

**Authors:** Henna Leskinen, Piia Lavikainen, Kari Linden, Mella Louhisalmi, Johanna Timonen

**Affiliations:** a School of Pharmacy, University of Eastern Finland, Yliopistonrinne 3, 70210 Kuopio, Finland; b University Pharmacy, P.O.Box 17, Valimotie 7, 00381 Helsinki, Finland

**Keywords:** Pharmaceutical waste, Expired medicines, Unnecessary medicines, Households, Disposal practices, Theory of Planned Behavior, Structural equation modeling

## Abstract

**Background:**

Growing medicine use raises environmental concerns due to pharmaceutical residues, which can be mitigated through proper household disposal.

**Objective(s):**

This study investigated household practices and background factors related to the proper disposal of unnecessary and expired medicines in Finland. Using the extended Theory of Planned Behavior (TPB), the study also explored psychosocial factors influencing individuals' intention and behavior regarding proper medicine disposal.

**Methods:**

An online survey was conducted among loyalty customers of the University Pharmacy in 2023. The data were analyzed using frequencies, Chi-Square tests, and Structural Equation Modeling (SEM) based on the extended TPB.

**Results:**

The final data included 5004 respondents. Most households reported returning unnecessary (84.7%) and expired (97.6%) medicines to pharmacies or designated collection points, while 33.5% retained unnecessary medicines for potential future use. Key factors influencing disposal practices included age, education, chronic illness, and recycling habits. SEM revealed that personal norms, perceived behavioral control, situational factors, and subjective norms significantly influenced disposal intentions, while intention, situational factors, and perceived behavioral control directly affected disposal behavior.

**Conclusions:**

While proper medicine disposal is common in Finnish households, further improvement is needed, particularly regarding unnecessary medicines. The findings highlight the need for ongoing communication and easy-to-use and time-effective disposal solutions. Communication should be designed to engage both the rational and emotional dimensions of medicine users and appeal to moral values, such as sense of duty and responsibility. Communication should specifically target groups less engaged in proper disposal, such as younger individuals and those without chronic conditions.

## Highlights

• Most households returned unnecessary and expired medicines properly to pharmacies.
• Retaining unnecessary medicines for potential future use was also common.
• Several background factors were associated with proper medicine disposal.
• Personal norms and perceived behavioral control most strongly influenced intention.
• Intention to properly dispose of medicines was the strongest predictor of behavior.

## 1 Introduction

Medicine use is expected to rise due to population growth and aging, the introduction of novel pharmaceuticals, and improved access.^1^ Although better access enhances health outcomes, it also increases environmental risks from pharmaceutical residues.^2^ The primary source of these residues is excretion through urine in wastewater, but improper household disposal − such as discarding medicines in mixed waste or sewage − also contributes.^3^ As this route is largely preventable, interest in promoting proper disposal practices is growing. The European Commission has addressed this issue in its Strategic Approach to Pharmaceuticals in the Environment.^4^

The current EU Directive 2004/27/EC obliges Member States to establish a collection system for unnecessary and expired household medicines, i.e., pharmaceutical waste.^5^ In most Member States, community pharmacies act as collection points.^6^ Outside the EU, pharmaceutical waste collection systems are generally less well organized, and national guidelines are often inadequate or entirely lacking.2., 7, 8, 9, 10, 11, 12, 13, 14 These collection systems are often based on voluntary participation, with pharmacies generally serving as designated collection points. In the United States, several methods for the disposal of pharmaceutical waste are recommended, including collection points, take-back events, and mail-back programs using designated return envelopes.15., 16

Several studies conducted in Asia,^8^, ^9^, ^17^ Africa,^7^, ^12^, ^18^, ^19^ America,^16^, ^20^ Australia,^21^ and Europe^22^, ^23^, ^24^, ^25^ indicate that medicines are most often disposed of improperly with household mixed waste. However, some studies conducted in Europe^26^, ^27^, ^28^, ^29^ and the United States^30^ have reported high rates of proper disposal. Among these, one Finnish study reported the highest return rates: approximately 90% of households returned such medicines to pharmacies.^28^ Previous studies also indicate that households tend to retain unnecessary medicines for future use, while expired ones are more often discarded.^7^, ^17^, ^26^, ^31^ In addition to environmental risk, improper disposal of medicines can compromise medication safety.^2^

National guidelines and differences in collection systems can influence household practices relating to the disposal of unnecessary and expired medicines.^29^, 32. Demographic factors, such as age, education, gender, and the presence of children, have also been linked to disposal practices.^19^, ^24^, ^25^, ^28^ Awareness of proper medicine disposal, risk perception, and knowledge of the environmental impacts of pharmaceuticals also play a role,^10^, ^25^, ^27^ though they only partially explain behavior.^10^ To deepen understanding, some studies have examined psychosocial factors influencing intention and behavior,^25^, ^33^, ^34^ though these have largely been conducted in countries where improper disposal is more common than returning medicines to pharmacies or other collection points.

The aim of this study was to examine disposal practices relating to unnecessary and expired medicines in Finnish households. While previous research has addressed disposal practices in Finland,^28^, ^35^ no studies have distinguished between unnecessary and expired medicines and the background factors influencing their disposal. This study investigated the association between respondents' demographic, health-related, and household recycling practices and proper medicine disposal. Using the extended Theory of Planned Behavior (TPB), it also examined which psychosocial factors influence individuals' intention and actual behavior in the proper disposal of medicines.

## 2 Extended Theory of Planned Behavior and hypotheses development

**Fig. 1:**
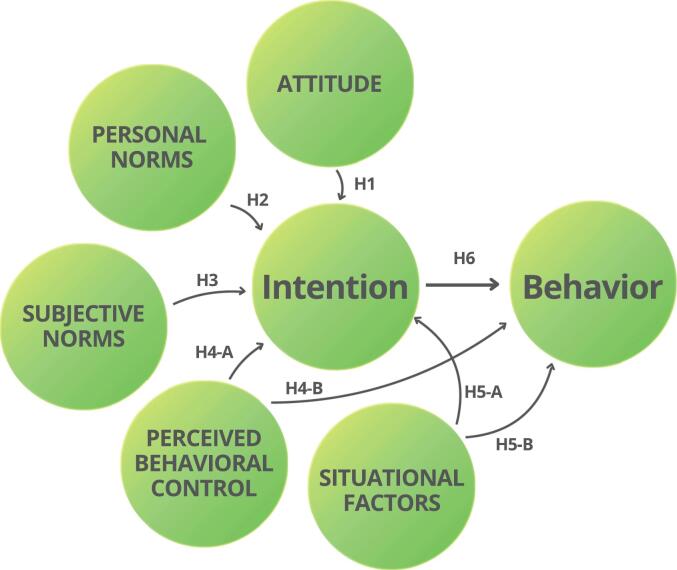
The theoretical model illustrating the factors that influence household medicine disposal intention and behavior within the extended Theory of Planned Behavior (TPB) framework. Ajzen's^36^ original model comprises attitude, subjective norms, perceived behavioral control, intention, and behavior, with personal norms and situational factors later added as extensions.

The Theory of Planned Behavior (TPB) provides a framework for understanding and predicting complex human behavior in a specific context.^36^ In the original model, behavior is influenced by intention (INT) and perceived behavioral control (PBC) (Fig. 1), while INT can be strengthened or weakened by attitude (ATT), subjective norms (SN), and PBC. Although each factor contributes to INT, their relative weights depend on behavior (BEH). TPB has been successfully applied in health behavior research (e.g. treatment adherence)^37^ and environmental behavior, including sorting and recycling and pro-environmental actions.^38^, ^39^, ^40^, ^41^ While TPB effectively predicts INT and BEH,36., 42 its explanatory power can be enhanced by incorporating additional variables, such as moral norms and situational factors.^43^, ^44^, ^45^ Ajzen^36^ acknowledged that in certain situations, adding moral or personal norms (PN) is necessary to capture an individual's moral obligation and sense of responsibility towards specific behaviors. In pro-environmental behavior, this means that individuals may prioritize environmentally responsible actions over perceived convenience. As a result, PN serve as a key factor alongside ATT and PBC when predicting pro-environmental INT.^46^ Similarly, situational factors (SF) can reflect the influence of an individual's objective environment on their INT and ability to engage in the final BEH, expanding TPB's ability to capture the non-motivational aspects of behavioral control.^33^, ^39^ Given that decisions regarding the proper disposal of medicines may involve behavioral, normative, and control factors, this study integrates PN and SF into the TPB framework (Fig. 1). Based on the extended TPB framework, ATT, SN, PBC, SF, and PN are expected to influence behavioral intention (INT), which in turn predicts proper medicine disposal behavior (BEH). In addition, PBC and SF may also directly influence behavior. The theoretical relationships between these factors, as well as the hypotheses of this study are described in more detail in the following section.

In the TPB framework, ATT refers to an individual's evaluation of a BEH based on its perceived advantages and disadvantages.^36^ Previous studies demonstrate that individuals generally consider the potential benefits and costs their behavior may have in terms of the environment.^47^ In the pro-environmental context, ATT has consistently predicted INT.^41^ These findings also align with medicine-related environmental ATT, where an individual's general environmental attitude plays a key role in shaping how they perceive the environmental effects of medicines.^48^ Previous research has identified ATT as a significant predictor of INT to properly dispose of household medicines.^33^, ^34^ These studies have typically operationalized ATT as evaluations of the proper disposal behavior itself, rather than broader environmental attitudes related to medicines. While defining specific behavior is essential within the TPB framework, it is also important to examine the role of ATT towards the environmental effects of medicines in shaping INT.

H1 ATT towards environmental effects of medicines positively affects an individual's intention to dispose of medicines properly.

PN refer to an individual's moral obligation to engage in or avoid a certain behavior.^49^ These norms are shaped by awareness of the consequences and a sense of personal responsibility for the outcomes of the individual's actions. In the pro-environmental context, PN are a strong predictor of INT and enhance the explanatory power of the TPB.^46^, ^50^ Previous studies have shown PN to significantly influence INT related to household waste separation, recycling,^39^, ^45^, ^51^ and proper medicine disposal.^33^

H2 PN positively affect an individual's intention to dispose of medicines properly.

SN refer to an individual's perception of social pressure to engage in a particular behavior.^36^ This perception is influenced by the expectations and opinions of significant others such as family members, friends, and the local community. SN have been shown to positively influence INT in various pro-environmental behaviors − for example, in household waste separation.^38^, ^39^ However, findings regarding medicine disposal are mixed: while some studies report a positive effect of SN on INT,^34^ others find no significant association.^25^, ^33^

H3 SN positively affect an individual's intention to dispose of medicines properly.

PBC refers to an individual's perception of how easy or difficult it is to engage in a particular behavior as shaped by past experiences and self-efficacy.^36^ PBC has a dual role in the TPB framework, as it plays a key role in predicting INT while also having a direct effect on BEH. Research on pro-environmental behavior^50^ and household waste sorting confirms this dual role.^52^ In the context of pharmaceuticals, PBC has been shown to positively affect INT to dispose of medicines properly^33^ and to reduce pharmaceutical waste in households.^53^

H4-a PBC positively affects an individual's intention to dispose of medicines properly.

H4-b PBC positively affects an individual's medicine-disposal behavior.

SF are closely related to PBC as they both reflect forms of control beliefs. They differ in scope, however: PBC reflects the individual's perceived ability to engage in the intended behavior, whereas SF refer to external, objective conditions that facilitate or hinder behavior.^43^ In recycling research, SF are typically measured by assessing individuals' perceptions of how time, space, access to facilities, and the cost of recycling will affect their actual recycling behavior.^38^, ^39^, ^43^ Zhang et al.^39^ observed a negative impact on waste separation BEH, suggesting that perceived obstacles can hinder recycling efforts. Foon et al.^33^ found that perceived busyness negatively affects the INT to dispose of medicines properly, highlighting that the perception of time availability is a key predictor.

H5-a SF negatively affect an individual's intention to dispose of medicines properly.

H5-b SF negatively affect an individual's medicine disposal behavior.

In the TPB, INT is a central factor that strongly predicts BEH.^36^ It reflects an individual's motivation and effort made to engage in a particular behavior. Although stronger INT generally increases the likelihood of BEH, actual performance may still depend on control-related factors, such as ability to overcome barriers or access necessary resources. Meta-analyses have consistently shown that pro-environmental INT strongly predicts actual BEH.^41^, ^46^, ^50^ However, Morren and Grinstein^41^ found that this relationship is stronger in developed countries than in developing countries. This can be explained by the greater economic capabilities of developed countries, which offer individuals access to better technologies and infrastructure, reducing barriers to acting in an environmentally friendly way. In recycling-related studies, the predictive power of INT on BEH has been mixed: Ghani et al.^38^ reported a weak effect in household food waste separation, whereas Huang et al.^51^ found a strong relationship in household waste sorting. Watkins et al.^25^ observed a moderate association between INT and proper medicine disposal.

H6 INT to dispose of medicines properly positively affects an individual's medicine disposal behavior.

## 3 Methods

### 3.1 Study context

Finland classifies all pharmaceutical waste as hazardous,^54^ and its disposal is regulated by the National Waste Act.^55^ Since the early 1990s, municipalities have been responsible for organizing the reception and management of household hazardous waste through contractual arrangements with waste treatment facilities and local pharmacies.56., 57. Under this contract, community pharmacies serve as collection points for unnecessary and expired medicines. As pharmacies are geographically well distributed across the country, with nearly every municipality having at least one pharmacy, households have convenient access to these collection points.^58^ In addition, medicines, except for nicotine replacement products, are only sold in pharmacies.^59^ Finland has a privately owned community pharmacy system. There are also two university owned pharmacies: the University Pharmacy of Helsinki and the University Pharmacy of Eastern Finland. The University Pharmacy of Helsinki is Finland's largest community pharmacy operator with 17 subsidiary pharmacies and an online pharmacy, serving over one million loyal customers with approximately six million customer interactions in its pharmacies and 20 million online visits annually.^60^

### 3.2 Data collection

This was a cross-sectional study conducted in Finland. Data was collected using an online questionnaire in June 2023. The questionnaire link was distributed via an email newsletter to all legal age (≥ 18 years) loyal customers of the University Pharmacy of Helsinki who had consented to receive research newsletters (*n* = 247,000). The questionnaire was open from June 13 to June 27, 2023, and no reminder was sent. However, the University Pharmacy posted a message on the messaging service X on June 14, 2023 to encourage participation in the study.

### 3.3 Questionnaire

The questionnaire (Appendix 1) consisted of 41 questions divided into six sections covering respondents' background, health-related information, and household medicine management practices. The questionnaire was designed for this study based on the objectives of the study and previous studies.^17^, ^28^, ^33^, 36., 38, 39, 48, 61, 62, 63 The questionnaire was conducted using the Webropol platform and piloted twice – first with research colleagues (*n* = 9) and laypersons (n = 9), leading to structural adjustments and clarification of key terms, and then with a second group (*n* = 10), after which no further modifications were necessary. No formal validity or reliability testing was conducted for the questionnaire.

This study focuses on findings from the final two sections of the questionnaire, which explored medicine disposal practices and perceptions of proper medicine disposal. Households' practices regarding the disposal of unnecessary or expired medicines were investigated with two structured questions “What do you do with unnecessary medicines in your household?” and “What do you do with expired medicines in your household?” with five predefined possible responses concerning disposal practices and an open ‘other’ field (questions 32–33, Appendix 1). The terms “unnecessary medicine” and “expired medicine” were defined in the questionnaire, and respondents were told that their responses should reflect the practices of all household members. The frequency of returns was assessed using the structured question “How often do you return unnecessary or expired medicines to the pharmacy in your household?” with six possible responses (question 34, Appendix 1).

Perceptions regarding proper medicine disposal were investigated using four Likert-scale questions (questions 38–41, Appendix 1), each comprising 6–10 statements designed to reflect latent factors in the extended TPB model influence the proper disposal of medicines (Table 1). As latent factors are variables that cannot be directly measured, the study used Likert-scale statements as reflective indicators to deduce the underlying latent constructs (attitude towards environmental effects of medicines (ATT), subjective norms (SN), personal norms (PN), perceived behavioral control (PBC), intention (INT), behavior (BEH), situational factors (SF)). The indicators for ATT were adapted from Alajärvi et al.,^48^ and indicators for SN, PN, PBC, SF, INT, and BEH were adapted from Ghani et al.,^38^ Zhang et al.,^39^ and Foon et al.,^33^ and the research group decided which of these were included in the present survey. Each indicator was measured on a 5-point Likert scale, with an “I don't know” option. Questions that elucidated the disposal practices for unnecessary or expired medicines (questions 32 and 33, Appendix 1) were also used to indicate respondents' proper disposal behavior.

**Table 1:** Questionnaire items of Likert-scale questions and their underlying latent factors. Measured with a 5-point scale: (1) Completely agree, (2) Somewhat agree, (3) Somewhat disagree, (4) Completely disagree, (5) I don't know.

Latent factor	Indicator^a^
Attitude towards environmental effects of medicines (ATT)	ATT1. Pharmaceutical residues in nature pose a risk to the environment
	ATT2. I am worried about the impact on human health of pharmaceutical residues in the environment
	ATT3. I am worried about the potential environmental impact of pharmaceutical products
	ATT4. The physician prescribing a medicine should consider the environmental impact of the product where possible
	ATT5. Leaflets in medicine packs describe the correct way to dispose of the medicine
	ATT6. Finland should set an example for other countries in reducing the impact of pharmaceutical products
Subjective norms (SN)	SN1. My family/close ones think I should dispose of medicines properly
	SN2. In my residential area, like others I am expected to dispose of medicines properly
	SN3. Taking medicines out of the home makes my house safer (for myself, family members, and pets)
Personal norms (PN)	PN1. I feel that it is my duty to dispose of medicines properly to protect the environment
	PN2. I feel (or would feel) guilty if I did not dispose of medicines properly
	PN3. I find it respectful and important that people dispose of medicines properly
Perceived behavioral control (PBC)	PBC1. I know where I can take the medicines for proper disposal
	PBC2. It is easy for me to take the medicines for proper disposal
	PBC3. I dispose of unnecessary medicines properly, regardless of whether I receive money for it (e.g. a deposit)
	PBC4. The proper disposal of medicines is simple
	PBC5. It is entirely my own decision whether or not I dispose of medicines properly
Intention (INT)	INT1. I am happy to dispose of medicines in accordance with the instructions
	INT2. I intend to dispose of medicines properly in the future
	INT3. I am happy to dispose of medicines properly if I know why it is worth doing so
	INT4. I regard myself as committed to disposing of medicines properly in the future
Behavior (BEH)	BEH1. So far, I have disposed of medicines properly (or I would have done so, had I had any medicines to dispose of)
	BEH2. I have not usually disposed of medicines properly^b^
	BEH3. I have been trying to reduce the amount of medicines in my home
	BEH4. What do you do with unnecessary medicines in your household?^c^
	BEH5. What do you do with expired medicines in your household?^c^
Situational factors (SF)	SF1. I do not have time to dispose of medicines in the proper way
	SF2. Difficulty going to the pharmacy or at the collection point hinders my proper disposal of medicines
	SF3. The fact that it is not mandatory hinders the proper disposal of medicines
	SF4. The cost of the medicine makes me less willing to dispose of it
	SF5. I have a safe place to store medicines^b^

a The indicators for ATT were adapted from reference Alajärvi et al.^48^ and indicators for SN, PN, PBC, SF, INT, and BEH were adapted from references Ghani et al.^38^, Zhang et al.^39^ and Foon et al.^33^.

b Reverse coded.

c Converted to 3-point scale: (1) pharmacy, (2) pharmacy and other disposal practice, (3) disposal practice other than the pharmacy.

For the background variables, respondents were asked structured questions about their year of birth, gender, region and area of residence, highest education level, health sector education (for both respondent and household members), household disposable income, health status, and chronic illnesses (questions 2–12, Appendix 1). Household size was asked with an open-ended question (question 4, Appendix 1). The question regarding the amount of household waste (other than medicines) that respondents sorted and recycled (question 37, Appendix 1) was also used as a background factor.

### 3.4 Data analysis

Data analysis was conducted in two phases. First, data were prepared and analyzed descriptively using IBM SPSS Statistics for Mac, version 29.0.2.0 (SPSS, Inc., Chicago, IL, USA). Group differences in categorical variables (gender, age, health status, chronic illnesses, education, health sector education, household type, region and area of residence, household income, sorting and recycling of household waste) were examined using Pearson's Chi-Square test, with *p*-values <0.05 considered statistically significant. During data preparation, continuous background variables (age and size of household) were converted into categorical variables, and health sector education for both respondent and household member were combined into one variable. Some response categories from the background variables region and area of residence, education, household disposable income, and health status were combined to ensure a sufficient number of observations in each category for the analyses. For the gender variable, the response options “other” and “prefer not to answer” were excluded from Chi-Square testing due to small group sizes. The questions concerning the disposal practices for unnecessary and expired medicines (questions 32 and 33, Appendix 1) included open-ended responses, which were either categorized under the five predefined response options on disposal practice or left under the “other” category. Subsequently, the six response options for these questions were merged into three categories to examine their association with background variables and to serve as indicators in the Structural Equation Modeling (SEM) analysis: (1) Pharmacy - exclusively pharmacy or other collection point for medicines selected; (2) Pharmacy and other - pharmacy or other collection point for medicines plus at least one other disposal practice selected; (3) Other - exclusively other disposal practice selected.

In the second phase of the data analysis, SEM was performed using Mplus, version 8 (Muthén & Muthén, Los Angeles, CA, USA) to test the theoretical model based on the extended TPB. SEM was selected for its ability to analyze complex relationships between observed variables (indicators) and latent constructs (factors), as well as to test hypothesized paths between exogenous and endogenous latent variables.^64^ In this study, the original model included seven latent factors and 31 indicators (Table 1), with INT and BEH specified as endogenous variables, and ATT, PN, SN, PBC, and SF as exogenous variables. SEM comprises two main components: the measurement model and the structural model.^65^ The measurement model involves assigning observed indicators to latent constructs and evaluating construct validity. The structural model then tests hypothesized relationships between latent variables, assessing the strength and direction of these paths. Given the use of an extended TPB framework and established indicators from previous research, Confirmatory Factor Analysis (CFA) was conducted to evaluate the measurement model, assessing the fit between indicators and their latent constructs prior to structural analysis. Results from the measurement model were reported as polychoric correlations between latent factors, fit indices, and standardized factor loadings.^66^ Fit indices were used to assess how well the hypothesized model fitted the empirical data.^65^ Absolute fit was assessed using the Root Mean Square Error of Approximation (RMSEA), Weighted Root Mean Square Residual (WRMR), and Chi-Square (χ^2^) test values. These indices assess how well the model replicates the observed data, with values closer to zero indicating a better fit. Incremental fit was assessed using the Tucker-Lewis Index (TLI) and Comparative Fit Index (CFI). Unlike absolute fit, these indices compare the hypothesized model's fit against a baseline model assuming uncorrelated observed variables. Values range from 0 to 1, with values closer to one indicating a better fit. Results from reliability and validity analyses were calculated and reported as average variance extracted (AVE), composite reliability (CR), Cronbach's alpha, and the heterotrait-monotrait ratio of correlations 2 (HTMT2) values.^65^, 67., 68

After validating the measurement model, the structural model and hypotheses were tested. Fit indices (TLI, CFI, RMSEA, WRMR, χ^2^), statistically significant (*p* < 0.05) standardized path coefficients, and the coefficient of determination (R^2^) were reported, forming the basis for the final structural model. All SEM analyses employed the weighted least squares mean and variance adjusted (WLSMV) estimator, which is suitable for categorical data that do not meet normality and continuity assumptions.^69^, ^70^ However, the WLSMV estimator has limitations with missing data, potentially producing biased estimates in the presence of data Missing at Random (MAR).^71^ In this study, the missing data ranged from 0.1% to 40.8% due to “I don't know” responses (Appendix 2). Little's MCAR test indicated that the missing data was not Missing Completely at Random (MCAR) (*p* < 0.001). Therefore, multiple imputation was performed before analysis to minimize potential bias in the estimates, following the recommendations of Asparouhov and Muthen,^71^ with 10 imputed datasets.

### 3.5 Ethical statement

The study followed national ethical principles for non-medical human sciences.^72^ Ethical approval was not required, as the study did not fall within the scope of research requiring prior ethical review in Finland. Participation in the study was voluntary, and informed consent was obtained from all participants at the beginning of the survey. The personal data collected was handled in compliance with the General Data Protection Regulation (EU) 2016/679^73^ and the Finnish Data Protection Act (1050/2018).^74^

## 4 Results

A total of 5030 respondents completed the questionnaire. After excluding 26 individuals who did not consent, the final dataset comprised 5004 respondents. The majority of the respondents were female (87.1%, *n* = 4358), with a mean age of 60.9 years (SD = 13.9, range = 18–95). More detailed characteristics of the study respondents are presented in Table 2.

**Table 2:** Characteristics of the study respondents (*n* = 5004).

Variable	n	%
**Gender**		
Female	4358	87.1
Male	578	11.6
Other	22	0.4
Prefer not to answer	46	0.9
**Age in years**		
18–34	305	6.1
35–59	1658	33.1
60–74	2270	45.4
≥ 75	771	15.4
**Health status**		
Good or fairly good	3423	68.4
Moderate	1320	26.4
Poor or fairly poor	261	5.2
**Chronic illness**		
Yes	4269	85.3
No	735	14.7
**Education**		
Elementary school^a^	461	9.2
Upper secondary school	2402	48.0
Tertiary education	2141	42.8
**Health sector education**		
Yes	1312	26.2
No	3692	73.8
**Type of household**		
Single household	1707	34.1
Other adult household	1919	38.3
Household with children	1348	26.9
Unknown	30	0.6
**Region of residence**		
Southern Finland	2676	53.5
Southwestern Finland	661	13.2
Western and Inland Finland	811	16.2
Eastern Finland	389	7.8
Northern Finland	338	6.8
Lapland	129	2.6
**Area of residence**		
Helsinki metropolitan area city center or suburb	1907	38.1
Other city center or suburb	2348	46.9
Town center, village, or urban area	383	7.7
Rural area	366	7.3
**Household income (euros)**		
≤ 2000	1071	21.4
2001–4000	1007	20.1
> 4000	2102	42.0
Do not know or do not want to answer	824	16.5
**Sorting and recycling of household waste (other than medicines)**		
All	2493	49.8
Almost all	2076	41.5
Some	413	8.3
None	22	0.4

a or education unknown.

### 4.1 Household practices regarding the disposal of unnecessary or expired medicines

Most households reported returning unnecessary (84.7%, *n* = 4238) and expired (97.6%, *n* = 4886) medicines to pharmacies or designated collection points, while 33.5% (*n* = 1677) retained unnecessary medicines for potential future use (Table 3). Most households (72.1%, *n* = 3609) reported returning unnecessary and expired medicines to the pharmacy at least once a year or more frequently.

**Table 3:** Disposal practices for unnecessary and expired medicines reported by the study respondents (*n* = 5004), and the frequency of returning medicines to a pharmacy.

Disposal practice for unnecessary medicines	n^a^	%^a^
Returned to a pharmacy or other collection point for medicines	4238	84.7
Retained for possible future use	1677	33.5
To be given for someone outside the household to use	158	3.2
Put in with mixed waste	75	1.5
Put down the drain	11	0.2
Other	33	0.7
**Disposal practice for expired medicines**		
Returned to a pharmacy or other collection point for medicines	4886	97.6
Retained for possible future use	254	5.1
To be given for someone outside the household to use	10	0.2
Put in with mixed waste	119	2.4
Put down the drain	16	0.3
Other	49	1.0
**Frequency of returning medicines to a pharmacy**		
Every 1–3 months	396	7.9
Every six months	1377	27.5
Once a year	1836	36.7
Every two years	448	9.0
Less frequently	856	17.1
Never	91	1.8

a In questions about the disposal practice for unnecessary or expired medicines, the respondents could choose several response options.

### 4.2 Association between background factors and disposal of unnecessary or expired medicines

Statistically significant differences in household disposal practices for unnecessary and expired medicines were associated with respondents' gender, age, health status, chronic illness, education level, household health sector education, and general waste sorting and recycling habits (Table 4). No statistically significant differences were observed with respect to region or area of residence, household income, or household type. Male respondents more often reported that their households returned unnecessary medicines exclusively to pharmacies than female respondents (*p* = 0.043) (Table 4). Households of female respondents more often returned expired medicines to pharmacies and used other disposal practices less frequently than those of male respondents (*p* < 0.001). Younger respondents (18–34 years) reported less frequently that their households returned unnecessary medicines to pharmacies than older age groups (*p* < 0.001). Older respondents more often reported that their households returned expired medicines exclusively to pharmacies (*p* < 0.001).

**Table 4:** Disposal practices reported by the study respondents for unnecessary and expired medicines by gender, age, health status, chronic illness, education, health sector education, and household waste recycling practices. The table reports differences only for those background variables for which a statistically significant difference (*p* < 0.05) was found^a^.

	Unnecessary medicines			Expired medicines		
	Pharmacy^b^ % (n)	Pharmacy and other^c^ % (n)	Other^c^ % (n)	Pharmacy^b^ % (n)	Pharmacy and other^c^ % (n)	Other^d^ % (n)
**All (*n* = 5004)**	64.1 (3210)	20.5 (1028)	15.3 (766)	91.9 (4601)	5.7 (285)	2.4 (118)
**Gender (*n* = 4936)**^e^						
Female	64.0 (2787)	20.9 (909)	15.2 (662)	92.8 (4044)	5.4 (234)	1.8 (80)
Male	68.9 (398)	16.8 (97)	14.4 (83)	87.9 (508)	6.7 (39)	5.4 (31)
Chi-Square	**p = 0.043**			**p < 0.001**		
**Age in years**						
18–34	27.2 (83)	39.3 (120)	33.4 (102)	76.1 (232)	13.4 (41)	10.5 (32)
35–59	52.2 (866)	27.7 (460)	20.0 (332)	88.3 (1464)	8.6 (142)	3.1 (52)
60–74	72.3 (1641)	15.5 (351)	12.2 (278)	95.4 (2166)	3.6 (81)	1.0 (23)
≥ 75	80.4 (620)	12.6 (97)	7.0 (54)	95.8 (739)	2.7 (21)	1.4 (11)
Chi-Square	**p < 0.001**			**p < 0.001**		
**Health status**						
Good or fairly good	63.5 (2172)	20.1 (688)	16.4 (563)	92.1 (3151)	5.6 (190)	2.4 (82)
Moderate	66.7 (881)	20.4 (269)	12.9 (170)	92.7 (1223)	5.4 (71)	2.0 (26)
Poor or fairly poor	60.2 (157)	27.2 (71)	12.6 (33)	87.0 (227)	9.2 (24)	3.8 (10)
Chi-Square	**p = 0.002**			**p = 0.040**		
**Chronic illness**						
Yes	66.0 (2819)	20.3 (865)	13.7 (585)	92.5 (3950)	5.6 (238)	1.9 (81)
No	53.2 (391)	22.2 (163)	24.6 (181)	88.6 (651)	6.4 (47)	5.0 (37)
Chi-Square	**p < 0.001**			**p < 0.001**		
**Education**						
Elementary school	85.0 (392)	10.0 (46)	5.0 (23)	96.5 (445)	2.4 (11)	1.1 (5)
Upper secondary education	68.4 (1642)	17.9 (429)	13.8 (331)	93.5 (2245)	4.5 (107)	2.1 (50)
	54.9 (1176)	25.8 (553)	19.2 (412)	89.3 (1911)	7.8 (167)	2.9 (63)
Tertiary education						
Chi-Square	**p < 0.001**			**p < 0.001**		
**Health sector education**						
Yes	65.5 (859)	21.4 (281)	13.1 (172)	93.8 (1230)	5.1 (67)	1.1 (15)
No	63.7 (2351)	20.2 (747)	16.1 (594)	91.3 (3371)	5.9 (218)	2.8 (103)
Chi-Square	**p = 0.034**			**p = 0.002**		
**Sorting and recycling of household waste (other than medicines)**						
All	68.6 (1709)	17.8 (445)	13.6 (339)	93.9 (2340)	4.6 (114)	1.6 (39)
Almost all	60.9 (1264)	23.2 (481)	15.9 (331)	91.7 (1904)	6.2 (128)	2.1 (44)
Some	54.5 (225)	24.0 (99)	21.5 (89)	83.3 (344)	9.9 (41)	6.8 (28)
None	54.5 (12)	13.6 (3)	31.8 (7)	59.1 (13)	9.1 (2)	31.8 (7)
Chi-Square	**p < 0.001**			**p < 0.001**		

a There were no statistically significant differences in terms of region or area of residence, income, or household type.

b Includes responses where exclusively “pharmacy or other collection point for medicines” is selected.

c Includes responses where “pharmacy or other collection point for medicines” and at least one other disposal practice is selected.

d Includes responses where exclusively disposal practice other than “pharmacy or other collection point for medicines” is selected.

e In the gender variable, the response options ‘other’ and ‘prefer not to answer’ were excluded from the Chi-Square test due to the small size of these groups.

Respondents with poor or fairly poor health less often reported that their households used pharmacies as the sole disposal practice for unnecessary (*p* = 0.002) and expired (*p* = 0.040) medicines than those in better health (Table 4). Respondents with a chronic illness more often reported that their households disposed of unnecessary (*p* < 0.001) and expired (p < 0.001) medicines exclusively through pharmacies than those without a chronic illness.

Households of lower-educated respondents more often used pharmacies exclusively to dispose of unnecessary (*p* < 0.001) and expired (p < 0.001) medicines than those of higher-educated respondents (Table 4). Households with a member educated in the health sector more frequently used pharmacies as the sole disposal practice for unnecessary (*p* = 0.034) and expired (*p* = 0.002) medicines than households without such educational background.

Households that sorted and recycled all or nearly all of their waste were more likely to return unnecessary (p < 0.001) and expired (p < 0.001) medicines exclusively to pharmacies, compared to those recycling only some or none (Table 4).

### 4.3 Factors in the extended theory of planned behavior affecting intention and behavior to dispose of medicines properly

#### 4.3.1 Assessment of the measurement model

Responses to the Likert-scale statements used as model indicators displayed a J-shaped distribution, with selections skewed towards one end of the scale (Appendix 2). The overall model fit was assessed using several fit indices from CFA (Appendix 3). The CFI (0.971) and TLI (0.966) values exceeded the commonly recommended threshold of 0.95 and 0.96, respectively, and the RMSEA (0.039) was well below the recommended cut-off value of 0.06, indicating a good model fit.^66^ The χ^2^ statistic (χ^2^ = 2023.09, df = 231) was statistically significant and the WRMR value (1.990) relatively high. However, the χ^2^ test is known to be highly sensitive to large sample sizes and often leads to rejection of otherwise well-fitting models when N is large.^75^ Moreover, WRMR has been shown to be less stable and less widely recommended as a primary fit index compared with CFI, TLI, and RMSEA. Therefore, consistent with current SEM reporting practices, model fit was primarily evaluated using approximate fit indices (CFI, TLI, RMSEA), which all indicated a good fit of the measurement model.

Convergent validity was assessed by examining factor loadings, reliability, and AVE values (Table 5). Following Hair et al.,^65^ a factor loading threshold of 0.6 was applied, as values above 0.7 are preferred but ≥0.6 are acceptable with large samples. Indicators ATT4, ATT5, PBC5, BEH3, BEH4, and SF5 (Table 1) were excluded due to loadings below this threshold. PBC3 was excluded due to a high cross-loading without theoretical justification for loading for multiple factors.^76^ INT3 with a loading of 0.592 (95% CI 0.553–0,630) was retained as it closely approached the threshold. The remaining indicators had factor loadings between 0.592 and 0.981 (*p* < 0.001), indicating moderate to strong associations with their latent constructs.

**Table 5:** Reliability and validity of the study constructs (*n* = 5004).

Construct	Indicator^a^	Standardized factor loading [CI 95%]	AVE	CR	Cronbach's alpha
Environmental attitude towards medicines (ATT)	ATT1	0.843 [0.814, 0.872]	0.694	0.899	0.880
	ATT2	0.883 [0.867, 0.900]			
	ATT3	0.923 [0.908, 0.938]			
	ATT6	0.659 [0.626, 0.692]			
Subjective norm (SN)	SN1	0.799 [0.760, 0.838]	0.564	0.794	0.748
	SN2	0.797 [0.768, 0.826]			
	SN3	0.647 [0.607, 0.688]			
Personal norm (PN)	PN1	0.914 [0.891, 0.937]	0.705	0.876	0.876
	PN2	0.719 [0.690, 0.748]			
	PN3	0.873 [0.841, 0.905]			
Perceived behavioral control (PBC)	PBC1	0.861 [0.821, 0.901]	0.799	0.923	0.910
	PBC2	0.893 [0.874, 0.911]			
	PBC4	0.927 [0.909, 0.945]			
Intention (INT)	INT1	0.928 [0.913, 0.943]	0.770	0.928	0.924
	INT2	0.978 [0.962, 0.994]			
	INT3	0.592 [0.553, 0,630]			
	INT4	0.954 [0.940, 0.968]			
Behavior (BEH)	BEH1	0.981 [0.966, 0.996]	0.731	0.889	0.883
	BEH2	0.771 [0.738, 0.798]			
	BEH5	0.797 [0.763, 0.830]			
Situational factors (SF)	SF1	0.925 [0.892, 0.958]	0.591	0.850	0.844
	SF2	0.794 [0.767, 0.822]			
	SF3	0.620 [0.581, 0.659]			
	SF4	0.702 [0.665, 0.738]			

AVE = average variance extracted, CR = composite reliability.

a The Likert statements related to the indicators are presented in Table 1.

Scale reliability was evaluated using Cronbach's alpha and CR. Cronbach's alpha values above 0.7 indicate acceptable and 0.8 good reliability.^77^ Six factors exceeded 0.8, while SN showed a slighlty lower value (α = 0.748), indicating acceptable but modest reliability (Table 5). This somewhat lower value may partly reflect the limited number of indicators used to measure SN. All CR values exceeded the 0.7 threshold, ranging from 0.794 to 0.928, indicating good internal consistency across constructs. Convergent validity was further supported by AVE values exceeding the recommended 0.5 threshold, ranging from 0.564 to 0.799. Together, these results indicate adequate reliability and convergent validity of the measurement model.

Polychoric correlation among latent factors ranged from −0.792 to 0.925 (Table 6). The strongest positive correlation (0.925) was between INT and BEH, suggesting possible discriminant validity concerns.^77^ To further evaluate discriminant validity, HTMT2 values were examined.^68^ All HTMT2 values were below the recommended threshold of 0.9 (Table 7), supporting adequate discriminant validity despite the relatively high INT − BEH correlation.

**Table 6:** Polychoric correlations of latent constructs.

Factor	ATT	SN	PN	PBC	INT	BEH	SF
ATT	**1.000**						
SN	0.502	**1.000**					
PN	0.759	0.722	**1.000**				
PBC	0.403	0.588	0.602	**1.000**			
INT	0.605	0.712	0.836	0.818	**1.000**		
BEH	0.471	0.584	0.673	0.814	0.925	**1.000**	
SF	−0.313	−0.448	−0.548	−0.787	−0.738	−0.792	**1.000**

ATT = Environmental attitude towards medicines, SN = Subjective norms, PN = Personal norms, PBC = Perceived behavioral control, INT = Intention, BEH = Behavior, SF = Situational factors.

**Table 7:** Discriminant validity of the model assessed with the heterotrait-monotrait ratio of correlations 2 (HTMT2) values (values <0.90 indicate acceptable discriminant validity between two constructs).

Factor	ATT	SN	PN	PBC	INT	BEH	SF
ATT							
SN	0.566						
PN	0.779	0.772					
PBC	0.437	0.654	0.611				
INT	0.629	0.763	0.833	0.797			
BEH	0.476	0.622	0.659	0.824	0.869		
SF	0.310	0.450	0.526	0.729	0.687	0.801	

ATT = Environmental attitude towards medicines, SN = Subjective norms, PN = Personal norms, PBC = Perceived behavioral control, INT = Intention, BEH = Behavior, SF = Situational factors.

#### 4.3.2 The structural equation model

Several fit indices were assessed to evaluate the structural fit of the model. The structural model fit was comparable to the measurement model, with minor differences observed in χ^2^ and WRMR (χ^2^ (df = 234) = 2061.202, CFI = 0.971, TLI = 0.966, RMSEA = 0.040, WRMR = 2.017) (Appendix 3). Based on these values and the previously discussed problems with χ^2^ and WRMR, the extended TPB model demonstrated a good fit with the data overall. The model explained 85.0% of the variance in INT and 85.1% in BEH, indicating a strong explanatory power of the model's latent factors (Appendix 3).

**Fig. 2:**
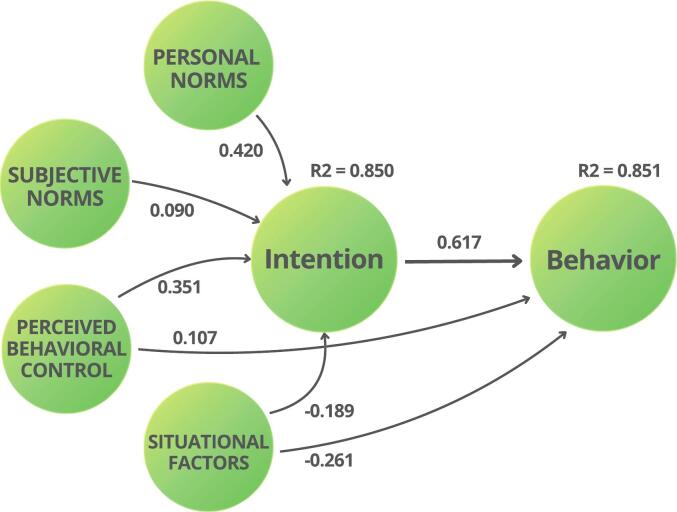
The estimated structural model from SEM analysis. Arrows indicate the direction of relationships, with values representing standardized path coefficients. R^2^ values represent the variance explained in the endogenous variables. Only statistically significant paths (*p* < 0.05) are displayed.

Path analysis results revealed that ATT did not significantly predict INT (*p* > 0.05), leading to the rejection of H1 (Appendix 4). SN (β = 0.090, 95% CI 0.002–0.178, *p* = 0.046), PN (β = 0.420, 95% CI 0.310–0.529, *p* < 0.001), and PBC (β = 0.351, 95% CI 0.264–0.438, p < 0.001) had significant positive effects on INT, with PN being the strongest predictor. SF had a negative effect on INT (ß = −0.189, 95% CI -0.269 to −0.108, p < 0.001) and BEH (ß = −0.261, 95% CI -0.329 to −0.193, p < 0.001). Furthermore, PBC (β = 0.107, 95% CI 0.017–0.197, *p* = 0.019) had a positive effect on BEH. INT (β = 0.617, 95% CI 0.548–0.687, p < 0.001) had the strongest significant positive effect on BEH. Based on the results, hypotheses H2, H3, H4-a, H4-b, H5-a, H5-b, and H6 were accepted, and H1 was rejected. The final structural model, including significant paths, is shown in Fig. 2.

## 5 Discussion

### 5.1 Household practices regarding the disposal of unnecessary or expired medicines

In this study, the majority of households disposed of unnecessary and expired medicines properly by returning them to pharmacies. Only a small portion disposed of them by putting them in mixed waste or put them down the drain. The finding is consistent with a previous Finnish study.^28^ The findings suggest that proper medicine disposal remains high in Finnish households and exceeds levels reported both in other European countries,^24^, ^25^, ^26^, ^27^, ^29^, 32. and also globally, where disposal via household waste or drainage is still very common.^9^, ^16^, ^17^, ^19^, ^21^ The high rate of proper medicine disposal in Finland may be attributed to the accessible, pharmacy-based take-back system. The system is free, long-established, and supported by legislation and public awareness, and over 90% of Finns report satisfaction with pharmacy availability.55., 56., 57., 78 Recent studies emphasize the need for context-specific collection schemes and improved consumer awareness to enhance proper medicine disposal.2., 8, 9, 32.

This study found differing disposal practices for unnecessary and expired medicines, largely due to households retaining unnecessary medicines for potential future use. Similar retention rates have been reported in previous studies,^8^, ^9^, ^12^, ^29^ with some noting that over half of households store unnecessary medicines for future use.^7^, ^17^, ^19^, ^26^, ^31^ Interestingly, some households also reported retaining expired medicines for future use – more often than disposing of them in mixed waste or via drainage. A similar trend was observed in Australia, where two-thirds of medicines kept for later use had expired.^21^

Retaining unnecessary or expired medicines in households contradicts Finnish guidelines, which recommend returning all unnecessary medicines to pharmacies regardless of their shelf life.^79^ Storing unnecessary or expired medicines may also pose medication safety risks,^2^ increase self-medication − particularly among young people^80^− and raises the risk of accidental ingestion by children or other household members.^81^ Finnish households typically store all medicines − regardless of use status or shelf life − together in unlocked spaces, most often in kitchen cupboards.^82^ These storage practices raise concerns about medication safety risks within the household. According to a previous study, households that return medicines less frequently tend to have a higher prevalence of expired medicines at home.^82^ In this study, most households returned unnecessary and expired medicines to pharmacies annually, which is consistent with other studies.^8^, ^23^ However, about one-quarter of households returned medicines only biannually or less, potentially contributing to the observed medicine retention.

### 5.2 Association between background factors and the disposal of unnecessary or expired medicines

Respondents' household disposal practices for unnecessary and expired medicines were significantly associated with several demographic factors. Consistent with previous studies,^24^, ^28^ improper disposal was more common among younger respondents, while proper disposal practices increased with age. Older individuals typically have more medicines in regular use and, due to Finland's reimbursement policy allowing three months' supply per purchase,^83^ visit pharmacies more frequently. These visits may increase opportunities to return unnecessary and expired medicines, potentially explaining age-related differences in disposal practices. Additionally, greater environmental awareness and concern about the impact of medicines on the environment among older people^48^ may contribute to higher rates of medicine returns. This study also supports previous findings indicating that individuals with chronic illnesses return medicines to collection points more frequently than those with acute conditions.^84^ As is the case with older adults, individuals with chronic illnesses visit pharmacies regularly, which may further increase the convenience of returning medicines during routine visits.

Respondents with a higher level of education reported more frequent use of improper disposal practices, particularly for unnecessary medicines, compared to those with a lower level of education. This finding contrasts with earlier studies suggesting that higher education is associated with proper disposal,^85^ attributed to better knowledge of safe disposal practices^86^ and greater awareness of the environmental impacts of medicines.^87^ However, these studies did not account for medicine retention as part of disposal practice, which may partly explain the discrepancy. Earlier studies have also shown that individuals with a higher level of education tend to have more unnecessary and expired medicines at home,^82^, ^84^ possibly due to greater health literacy,^88^ which may encourage them to more actively self-medicate, resulting in more medicines at home. Furthermore, higher education is associated with better overall health and a lower incidence of chronic illnesses,^89^ which may reduce pharmacy visit frequency and make medicine returns less routine and more inconvenient.

Sorting and recycling all or most general waste was strongly associated with returning medicines primarily to pharmacies, which is consistent with previous findings.^28^ This association is likely influenced by shared determinants such as proximity to disposal sites, time investment, environmental awareness, and general environmental attitudes.^33^, ^48^, ^90^ Notably, respondents in this study reported higher levels of household waste recycling than the Finnish national average in 2022, when 43% of municipal waste was recycled.^91^ As Finland aims to increase the municipal waste recycling rate to 65% by 2035 in accordance with EU targets, improved recycling infrastructure and awareness campaigns may also promote proper pharmaceutical waste disposal.

### 5.3 Factors in the extended theory of planned behavior affecting intention and behavior to dispose of unnecessary and expired medicines properly

PN, PBC, SF, and SN significantly affected the INT to dispose of medicines properly. Actual disposal BEH was influenced by INT, PBC, and SF. These factors explained a substantial proportion of the variance in both INT and BEH, demonstrating the model's strong explanatory power in predicting proper disposal of unnecessary and expired medicines, which is consistent with previous research.^33^, ^34^ Among the predictors, PN and PBC had the strongest positive effect on INT, which in turn significantly predicted BEH.

PN had the strongest positive affect on INT, which is consistent with previous findings.^33^, ^53^ In contrast, SN had only a weak effect on INT, despite both SN and PN reflecting normative beliefs. Similar findings have been reported in previous studies, with some even identifying SN as an insignificant predictor.^25^, ^33^ These findings suggest that moral beliefs may play a more influential role than perceived social expectations in shaping household medicine disposal behavior.

In line with previous research on pharmaceutical waste^33^, ^53^ and household recycling behavior,^39^, ^40^, ^92^ PBC significantly influenced both INT and BEH in this study. However, contrasting results have been reported, with Xu et al.^34^ finding no significant effect, and Watkins et al.^25^ observing a negative association with proper medicine disposal. Variations in national collection systems may partly explain these varying results. In countries lacking accessible collection points or public awareness of them, individuals may perceive proper disposal as difficult or infeasible.^93^ Although Finnish households generally consider medicine disposal to be relatively easy (Appendix 2), this study underscores the need for further development of practical solutions, particularly for those people still engaging in improper disposal. Earlier research has shown that SF such as time constraints or inconvenient waste separation facilities can influence medicine disposal INT^33^ or waste separation BEH.^39^, ^92^ Consistent with these findings, SF in this study had a negative effect on both INT and BEH, with a stronger direct impact on BEH. It appears that limited time or difficulties accessing pharmacies still pose barriers for some individuals.

Contrary to the present hypothesis and earlier findings,^25^, ^33^, ^34^, ^53^ ATT did not significantly predict INT regarding proper medicine disposal. In this study, ATT was conceptualized more broadly as an attitude towards the environmental effects of medicines, rather than the attitude towards disposing of medicines, which may explain the divergence from earlier findings,^25^, ^33^, ^34^, ^53^ as a more specific measurement of ATT may be required to observe a significant effect on INT.36., 94 However, earlier research has also shown that general environmental attitudes are associated with proper medicine disposal practices among Finnish households,^28^ while the impact of attitudes towards the environmental effects of medicines has not been previously examined. Despite Finnish households' high awareness of the environmental impacts of pharmaceuticals and pro-environmental attitudes,^48^ ATT did not predict INT to dispose of medicines properly in this study. This may be due to the overriding influence of other factors, such as PN. In Finland, people have high trust in institutions and generally tend to follow the instructions given,^95^ which may strengthen the sense of personal responsibility to follow the official guidelines, diminishing the effect of ATT.

INT to properly dispose of medicines was the strongest predictor of BEH. This finding aligns with the TPB,^36^ research on proper medicine disposal,^25^ and household waste separation and recycling,^39^, ^51^, ^92^ where INT is a key determinant of BEH. In addition to this, a high polychoric correlation between INT and BEH indicates that these constructs are strongly related. However, the path coefficient from INT to BEH indicates that INT does not fully explain the variance in medicine disposal BEH. SF and PBC also contribute significantly to explaining the variance in BEH. Compared to previous studies,^33^, ^34^ this study explained a considerably greater variance in INT. This may be explained by the inclusion of SF compared to Foon et al.,^33^ and a completely different set of factors used in the study by Xu et al.^34^ According to this study, INT is a central factor in predicting proper medicine disposal BEH, so interventions targeting factors that strengthen it (PN, PBC, SF, and SN) will also positively influence proper medicine disposal.

### 5.4 Utilization of the results

This study enhanced understanding of household medicine disposal, particularly of unnecessary medicines, and identified background and psychosocial factors influencing disposal practices. To our knowledge, this is the first TPB-based study in a context where proper medicine disposal is common. The results help address a research gap and support the EU's Strategic Approach to Pharmaceuticals in the Environment^4^ by offering insight into improving collection systems and promoting responsible disposal practices in households.

Despite the general awareness in Finland of proper medicine disposal and the environmental impact of pharmaceuticals,^48^, ^87^ ongoing communication is needed to reduce household storage of unnecessary and expired medicines. The findings will assist the authorities and pharmacies in developing communication and educational campaigns on proper medicine disposal. These strategies should engage both the emotional and rational dimensions of medicine users to enhance their effectiveness. Communication should emphasize the individual's moral and ethical responsibility, as well as the health and environmental risks associated with the storage of unnecessary and expired medicines in households. Practical solutions are also needed to facilitate medicine disposal in all households by removing barriers such as limited access to collection points or time restrictions, and these solutions should be effectively communicated to medicine users. For instance, could expanding medicine-waste collection bins beyond pharmacies, introducing collection services, or providing mail-back envelopes facilitate appropriate and safe disposal of medicine waste? Furthermore, communication should continue to target all medicine users, with particular emphasis on the groups identified in this study as currently less engaged in proper disposal. Information should be provided through easily accessible communication channels and these should be adapted according to the target group, as different groups have different preferences.^87^ For example, young people prefer to receive pharmaceuticals-related environmental information via the internet. The role of healthcare professionals, particularly pharmacists working in community pharmacies, should be strengthened in communication in light of their direct contact with medicine users. They can also support this by promoting rational medicine use and medication adherence, which can contribute to improved health outcomes and reduced amounts of unnecessary and expired medicines in households.^82^, ^96^, ^97^ Finally, more research is needed on the effectiveness and impact of different communication and education strategies and interventions on medicine disposal practices and related behaviors. In addition, to support the development of practical solutions, research is needed to determine which take-back systems most effectively and cost-effectly promote proper disposal and how disposal practices evolve in response to system changes.

### 5.5 Strengths and limitations

The questions were not formally validated but were developed based on the objectives of the study, previous studies^17^, ^28^, ^33^, 36., 38, 39, 48, 61, 62, 63 and researcher expertise. The clarity and comprehensibility of the questions were tested by both researchers and laypersons, which enhanced content validity. As the survey relied on self-reported data, both the reliability and validity of the results may be affected. First, some respondents may have reported only their own disposal practices instead of those of the entire household. Second, behavior was measured through self-declared actions, which may not accurately reflect actual behavior due to social and moral desirability bias. On the other hand, the survey was answered anonymously, which may have reduced this bias. As the study used cross-sectional data, the results can only show an association between factors and not causality.

One strength of the study was the large sample size of Finnish medicine users aged 18 to 95. However, the online questionnaire and recruitment via email newsletter may have introduced selection bias. The study invitation was sent to a large number of loyal customers of the University Pharmacy Helsinki, but only a small proportion of them responded (2%), even though the final number of respondents was still large. This may indicate that those individuals with a stronger interest in environmental pharmaceutical issues may have been more likely to participate. In addition, those who do not use electronic services were excluded from the study. However, their proportion of the Finnish population is small.^98^ It is also typical in Finnish online surveys that individuals with higher education respond more actively than others,^48^, ^99^ which was also reflected in this study. Furthermore, as the target group was pharmacy customers, and thereby medicine users and their households, the findings are not generalizable to all Finnish households. Moreover, women were overrepresented compared to the general population of medicine reimbursement recipients.^83^ On the other hand, research has shown that women tend to play a greater role in managing health related matters within their household,^100^, ^101^ and they also respond to medicine related surveys in Finland more actively than men.^48^, ^99^ However, caution is warranted when generalizing the results to all medicine users. Moreover, as the study was conducted in Finland, where the pharmaceutical waste collection system and cultural practices may differ from those in other countries, the findings should be applied elsewhere with consideration given to these contextual differences. Therefore, future research would benefit from obtaining data on medicine disposal practices and the factors related to medicine disposal behavior from a more diverse study population and across different contextual environments. This would allow researchers to examine whether the findings are replicable in various populations and whether the relationships between predictor variables and outcomes remain consistent across different contexts. Such knowledge could deepen understanding of the complex issues related to medicine disposal and support the applicability of the results, for example in developing interventions aimed at promoting environmentally responsible behavior.

Although the study was based on the widely used extended Theory of Planned Behavior and extended with situational factors (SF) and personal norms (PN),^33^, 36., 38, 39 the model has certain limitations. The relatively high correlation between self-reported intention and behavior suggests a close relationship between these factors, and it is possible that respondents did not clearly distinguish between their intended and actual behavior in their responses. On the other hand, a small gap between intention and behavior may indicate effective implementation across all behavior-predicting factors. Future research could address this by employing study designs in which intention and behavior are measured at different time points. While the extended model explained a substantial amount of variance in intention and behavior, other factors may also influence proper medicine disposal. Therefore, to capture this complex behavior, future research should consider more comprehensive frameworks to gain a deeper understanding of the factors influencing proper disposal of household medicines.

## 6 Conclusion

This study indicates that most Finnish households dispose of unnecessary and expired medicines properly by returning them to pharmacies. However, unnecessary medicines are often stored for potential future use, highlighting room for improvement. Key factors influencing disposal practices included age, the presence of chronic illnesses, education level, and household recycling practices. The extended TPB model effectively captured the determinants of proper disposal: PN and PBC were the strongest predictors of INT, which in turn strongly influenced BEH. SF and PBC also had direct effects on BEH. These findings highlight the need for ongoing communication and education on proper medicine disposal, particularly concerning unnecessary medicines. Effective communication should be designed to engage both the emotional and rational dimensions of medicine users and appeal to moral values, such as sense of duty and responsibility. Easily accessible, user-friendly, and time-efficient disposal solutions are also needed, and these should be clearly communicated to medicine users. Communication should specifically target groups less engaged in proper disposal, such as younger individuals and those without chronic conditions.

## Declaration of generative AI and AI-assisted technologies in the manuscript preparation process

During the preparation of this work, the authors used Microsoft Copilot and Grammarly in order to assist with translating the questionnaire and improving the readability and language of the text and condensing the content. After using these tools, the authors reviewed and edited the content as needed and take full responsibility for the content of the published article.

## CRediT authorship contribution statement

**Henna Leskinen:** Writing – review & editing, Writing – original draft, Visualization, Methodology, Investigation, Formal analysis, Data curation. **Piia Lavikainen:** Writing – review & editing, Formal analysis. **Kari Linden:** Writing – review & editing, Supervision, Resources, Methodology, Investigation, Conceptualization. **Mella Louhisalmi:** Writing – review & editing, Supervision, Methodology, Investigation, Formal analysis, Data curation, Conceptualization. **Johanna Timonen:** Writing – review & editing, Supervision, Resources, Project administration, Methodology, Investigation, Funding acquisition, Conceptualization.

## Funding

This work was supported by the Social Insurance Institution of Finland (SII) [grant number 38/26/2021]. The funder had no role in the design of the study; the collection, analysis, or interpretation of data; the writing of the manuscript; or the decision to submit the article for publication. The opinions presented in this publication are solely those of the authors and should not be construed as reflecting the official position of the funding body.

## Declaration of competing interest

The authors declare the following financial interests/personal relationships which may be considered as potential competing interests:

Johanna Timonen reports financial support was provided by The Social Insurance Institution of Finland. Kari Linden reports a relationship with Viatris Oy that includes: speaking and lecture fees. Kari Linden reports a relationship with The Finnish Society for Rheumatology that includes: speaking and lecture fees. Kari Linden reports a relationship with The Pharmaceutical Learning Centre that includes: speaking and lecture fees. Kari Linden reports a relationship with Pfizer Oy that includes: speaking and lecture fees. If there are other authors, they declare that they have no known competing financial interests or personal relationships that could have appeared to influence the work reported in this paper.
