# Supplementary material for: Disposal of unnecessary and expired medicines in households: Practices and factors associated with proper medicine disposal using the extended Theory of Planned Behavior
Source: Explor Res Clin Soc Pharm. 2026 May 12;23:100799. doi: 10.1016/j.rcsop.2026.100799 (PMC13197783; doi:10.1016/j.rcsop.2026.100799)
Supplement: Supplementary file 1 — Supplementary material [file mmc1.docx]

**Supplementary material**

**Appendix 1**. The study questionnaire: Survey on Household Medicines (Note: The survey was conducted electronically using the Webropol software, and this is the Word version of the questionnaire)

1. I confirm that by answering this questionnaire and clicking the submit button, I give consent for my responses to be used for research purposes.
   1. Yes
   2. No

BACKGROUND INFORMATION

First some background information about you.

1. What is your year of birth?
   1. Dropdown menu: 1920–2005
2. Your gender?
   1. Female
   2. Male
   3. Other
   4. Prefer not to answer
3. How many persons currently live in your household (including yourself)? Write your answer in numbers.

____Adults

____Aged 13–17

____Aged 7–12

____Under 7

1. Which region do you live in?
   1. South Karelia
   2. South Ostrobothnia
   3. South Savo
   4. Kainuu
   5. Kanta-Häme
   6. Central Ostrobothnia
   7. Central Finland
   8. Kymenlaakso
   9. Lapland
   10. Pirkanmaa
   11. Ostrobothnia
   12. North Karelia
   13. North Ostrobothnia
   14. North Savo
   15. Päijät-Häme
   16. Satakunta
   17. Uusimaa
   18. Southwest Finland
2. Your current area of residence?
   1. Helsinki metropolitan area (Helsinki, Espoo, Vantaa, Kauniainen) city centre or suburb
   2. Other city centre or suburb
   3. Town centre, village, or urban area
   4. Sparsely populated area or countryside
3. What is your highest level of education?
   1. Basic education
   2. Upper secondary education
   3. Vocational education and training
   4. University of Applied Sciences
   5. University or higher education institution
   6. Other
4. Do you have any education in the health sector?
   1. Yes
   2. No
5. Has anyone else living in your household had education in the health sector?
   1. Yes
   2. No
   3. No other members in the household
6. What is the combined disposable income of the members of your household on average per month (all income after tax, i.e. net income)? If you don’t know exactly, please give your best estimate
   1. Up to €1000
   2. €1001–2000
   3. €2001–3000
   4. €3001–4000
   5. €4001–5000
   6. €5001–8000
   7. over €8000
   8. I don’t know
   9. I prefer not to answer

HEALTH AND USE OF MEDICINES

The following questions are about **your** health.

1. How is your current state of health?
   1. Good
   2. Fairly good
   3. Moderate
   4. Fairly poor
   5. Poor
2. Do you currently have any long-term illnesses diagnosed by a physician for which you are taking a prescription medicine? You can choose several options.
   1. Allergy
   2. Asthma
   3. Another respiratory disease
   4. Diabetes
   5. High blood pressure (hypertension)
   6. High blood cholesterol levels
   7. Heart disease
   8. Cancer
   9. Rheumatism
   10. Musculoskeletal complaints or disabilities (other than rheumatism)
   11. Depression
   12. Mental health problem (Other than depression)
   13. Skin disease
   14. Gastrointestinal disease
   15. Hypothyroidism or other disorder of the thyroid gland
   16. Eye disorders
   17. Epilepsy
   18. Migraine or other chronic headache
   19. Other long-term illness
   20. I don’t know
   21. No long-term illnesses for which I use prescription medicines

[When you select any of the aforementioned long-term illnesses (1–19), the following question will appear for each illness selected. The name of the illness in question will be displayed in the coloured area.]

1. How well have the prescription medicines you have used to treat your long-term illness helped?
   1. Well
   2. Fairly well
   3. Moderately well
   4. Rather poorly
   5. Poorly

HOUSEHOLD MEDICINES

The following question apply to all medicines in your household.

Please give the total number of medicines for all individuals and any pets that are part of your household. This includes medicines that are currently in use, unnecessary, or have expired, whether they are prescription, or over-the-counter medicines obtained from a pharmacy. The medicine packs may be opened or unopened.

1. What is the total number of medicine packs in your household?
   1. Dropdown menu 0–100

The following questions are about the medicines in use in your household. Please include all the medicines in use belonging to any individuals or pets in your household. The medicine packs may be opened or unopened.

A medicine in use refers to any medicine that is taken regularly or as needed.

1. How many prescription medicine packs are in use in your household?
   1. Dropdown menu 0–100
2. How many over-the-counter medicine packs obtained from a pharmacy are in use in your household?
   1. Dropdown menu 0–100

The following questions are about the unnecessary medicines in your household. Please include all the unnecessary medicines belonging to any individual or pet in your household. The medicine packs may be opened or unopened.

An unnecessary medicine refers to any medicine that has been left unused or is unlikely to be used again. However, the medicine should not have yet expired. (Questions about expired medicines will be asked on the following page.)

1. How many unnecessary prescription medicine packs are there in your household?
   1. Dropdown menu 0–100
2. How many unnecessary over-the-counter medicine packs obtained from a pharmacy are there in your household?
   1. Dropdown menu 0–100
3. What unnecessary medicines are there in your household? You can choose several options.
   1. Allergy medicines
   2. Asthma medicines
   3. Chronic obstructive pulmonary disease (COPD) medicines
   4. Other lung disease medicines
   5. Antibiotics
   6. Insulin
   7. Diabetes medicines (excluding insulin)
   8. Antihypertensive medicines
   9. Anticoagulants
   10. Cholesterol medicines
   11. Heart medicines
   12. Antirheumatics
   13. Migraine medicines
   14. Analgesics
   15. Cancer medicines
   16. Antidepressants
   17. Medicines for mental health issues (other than depression)
   18. Sleeping pills
   19. ADHD medicines
   20. Antiepileptics
   21. Heartburn medicines
   22. Antiemetics
   23. Contraceptives
   24. Estrogen replacement therapies
   25. Prostate medicines
   26. Thyroid medicines
   27. Medicines for eye disease
   28. Medicines for skin diseases (ointments or tablets)
   29. Antidiarrheals
   30. Laxatives
   31. Antifungal medicines
   32. Veterinary medicines
   33. Other medicine, what?_______
   34. There are no unnecessary medicine packs in my household

The following questions are about the expired medicines in your household. Please include all expired medicines belonging to any individuals and pets in your household. The medicine packs may be opened or unopened.

An expired medicine refers to a medicine whose shelf life has been exceeded. The shelf life indicates the date by which the medicine should be used before it expires. This expiration date is marked on the medicine pack.

1. How many expired prescription medicine packs are there in your household?
   1. Dropdown menu 0–100
2. How many expired over-the-counter medicine packs obtained from a pharmacy are there in your household?
   1. Dropdown menu 0–100
3. What expired medicines are there in your household? You can choose several options.
   1. Allergy medicines
   2. Asthma medicines
   3. Chronic obstructive pulmonary disease (COPD) medicines
   4. Other lung disease medicines
   5. Antibiotics
   6. Insulin
   7. Diabetes medicines (excluding insulin)
   8. Antihypertensive medicines
   9. Anticoagulants
   10. Cholesterol medicines
   11. Heart medicines
   12. Antirheumatics
   13. Migraine medicines
   14. Analgesics
   15. Cancer medicines
   16. Antidepressants
   17. Medicines for mental health issues (other than depression)
   18. Sleeping pills
   19. ADHD medicines
   20. Antiepileptics
   21. Heartburn medicines
   22. Antiemetics
   23. Contraceptives
   24. Estrogen replacement therapies
   25. Prostate medicines
   26. Thyroid medicines
   27. Medicines for eye disease
   28. Medicines for skin diseases (ointments or tablets)
   29. Antidiarrheals
   30. Laxatives
   31. Antifungal medicines
   32. Veterinary medicines
   33. Other medicine, what?______
   34. There are no expired medicine packs in my household
4. Why are some of your household medicines unnecessary or expired? You can choose several options.
   1. Condition/symptom improved
   2. Switched to another medicine
   3. Medicine caused side-effects
   4. Medicine not sufficiently effective
   5. Taking the medicine was uncomfortable
   6. Medicine was bought as a precaution
   7. Medicine pack unnecessarily large
   8. Limited shelf life of medicine after opening
   9. Medicine user felt no need for the medicine
   10. The user of medicine has moved away or died
   11. Other, what?_________
   12. There are no unnecessary or expired medicine packs in my household

[Answer the following question if you have selected options 1, 2, 3, 4, or 5 in the previous question. The chosen reason is marked in the coloured area.]

1. When answer from the previous question, who made the decision to discontinue the medicine? You can choose several options.
   1. The user of the medicine
   2. Physician
   3. Nurse
   4. Pharmacist
   5. Someone else, who?

STORAGE OF MEDICINES

The following questions are about the storage of medicines in your household. Please continue to include the medicines of all individuals and pets in your household when answering the questions.

1. In which room in your household are medicines stored? You can choose several options.
   1. The toilet
   2. The bathroom
   3. The utility room
   4. The wardrobe
   5. The kitchen
   6. The bedroom
   7. The living room
   8. The hallway
   9. Other, where? ______
2. In what place in your household are medicines stored? You can choose several options.
   1. In a medicine cabinet
   2. In the fridge
   3. In a cupboard
   4. In a drawer
   5. On a shelf
   6. On a table
   7. Other, where? _____
3. Is the place where the medicines are stored locked or otherwise secured so that children and/or pets cannot have access to the medicines?
   1. Yes
   2. No
   3. Some medicines yes and some no
4. Are expired medicines stored separate from other medicines in the household?
   1. Yes
   2. No
   3. Some are stored some are not

[Answer the following three questions if you answered 1 or 3 in the previous question]

1. In which room in your household are expired medicines stored? You can choose several options.
   1. The toilet
   2. The bathroom
   3. The utility room
   4. The wardrobe
   5. The kitchen
   6. The bedroom
   7. The living room
   8. The hallway
   9. Other, where? ______
2. In what place in your household are expired medicines stored? You can choose several options.
   1. In a medicine cabinet
   2. In the fridge
   3. In a cupboard
   4. In a drawer
   5. On a shelf
   6. On a table
   7. Other, where? _____
3. Is the place where the expired medicines are stored locked or otherwise secured so that children and/or pets cannot have access to the medicines?
   1. Yes
   2. No
   3. Some medicines yes some no

PRACTICES REGARDING MEDICINES

The following questions are about the practices adopted by persons in your household regarding the handling of medicines. An unnecessary medicine refers to any medicine that has been left unused or is unlikely to be used again. However, the medicine should not have yet expired. An expired medicine refers to a medicine whose shelf life has been exceeded. The shelf life indicates the time by which the medicine should be used before it expires. This expiration date is marked on the medicine pack.

1. What do you do with unnecessary medicines in your household? You can choose several options.
   1. Retained for possible future use
   2. To be given for someone outside the household to use
   3. Put in with mixed waste
   4. Put down the drain
   5. Return to a pharmacy or other collection point for medicines
   6. Other, what?____
2. What do you do with expired medicines in your household? You can choose several options.
   1. Retained for possible future use
   2. To be given for someone outside the household to use
   3. Put in with mixed waste
   4. Put down the drain
   5. Return to a pharmacy or other collection point for medicines
   6. Other, what? _____
3. How often do you return unnecessary or expired medicines to the pharmacy in your household?
   1. Every 1–3 months
   2. Every six months
   3. Once a year
   4. Every two years
   5. Less frequently
   6. Never

The following questions are about your personal habits.

1. Do you usually check the expiration date (the date by which the medicine should be used) before purchasing a medicine?
   1. Yes
   2. No
2. Do you usually check the expiration date (the date by which the medicine should be used) before using a medicine?
   1. Yes
   2. No

Well done, you are now well over halfway through! Thank you for taking the time to complete the survey.

Sincerely, the study team

PROPER DISPOSAL OF MEDICINES

The last four sets of questions are about the proper disposal of medicines**.**

Proper medicine disposal refers to taking unnecessary or expired medicines to a pharmacy or other collection point.

1. How much of your household waste (excluding medicines) do you sort and recycle?
   1. All
   2. Almost all
   3. Some
   4. None
2. What is your opinion on the following environmental statements relating to medicines? For each statement, please select the option you consider most appropriate.

|  | Completely agree | Somewhat agree | Somewhat disagree | Completely disagree | I don’t know |
| --- | --- | --- | --- | --- | --- |
| Pharmaceutical residues in nature pose a risk to the environment | 1 | 2 | 3 | 4 | 5 |
| I am worried about the impact on human health of pharmaceutical residues in the environment | 1 | 2 | 3 | 4 | 5 |
| I am worried about the potential environmental impact of pharmaceutical products | 1 | 2 | 3 | 4 | 5 |
| The physician prescribing a medicine should consider the environmental impact of the product where possible | 1 | 2 | 3 | 4 | 5 |
| Leaflets in medicine packs describe the correct way to dispose of the medicine | 1 | 2 | 3 | 4 | 5 |
| Finland should set an example for other countries in reducing the impact of pharmaceutical products | 1 | 2 | 3 | 4 | 5 |

1. What is your opinion on the following statements regarding your personal medicine disposal habits? For each statement, please select the option you consider most appropriate.

|  | Completely agree | Somewhat agree | Somewhat disagree | Completely disagree | I don’t know |
| --- | --- | --- | --- | --- | --- |
| I know where I can take the medicines for proper disposal | 1 | 2 | 3 | 4 | 5 |
| It is easy for me to take the medicines for proper disposal | 1 | 2 | 3 | 4 | 5 |
| So far, I have disposed of medicines properly (or I would have done so, had I had any medicines to dispose of) | 1 | 2 | 3 | 4 | 5 |
| I regard myself as committed to disposing of medicines properly in the future | 1 | 2 | 3 | 4 | 5 |
| I have not usually disposed of medicines properly | 1 | 2 | 3 | 4 | 5 |
| I have been trying to reduce the amount of medicines in my home | 1 | 2 | 3 | 4 | 5 |

The last two sets of questions concern your and your close circle’s attitude towards proper medicine disposal.

1. What you think about the following statements? For each statement, please select the option you consider most appropriate.

|  | Completely agree | Somewhat agree | Somewhat disagree | Completely disagree | I don’t know |
| --- | --- | --- | --- | --- | --- |
| I am happy to dispose of medicines in accordance with the instructions | 1 | 2 | 3 | 4 | 5 |
| I intend to dispose of medicines properly in the future | 1 | 2 | 3 | 4 | 5 |
| I am happy to dispose of medicines properly, if I know why it is worth doing so | 1 | 2 | 3 | 4 | 5 |
| I dispose of unnecessary medicines properly, regardless of whether I receive money for it (e.g. a deposit) | 1 | 2 | 3 | 4 | 5 |
| The proper disposal of medicines is simple | 1 | 2 | 3 | 4 | 5 |
| I do not have time to dispose of medicines in the proper way | 1 | 2 | 3 | 4 | 5 |
| Difficulty going to the pharmacy or at the collection point hinders my proper disposal of medicines | 1 | 2 | 3 | 4 | 5 |
| The fact that it is not mandatory hinders the proper disposal of medicines | 1 | 2 | 3 | 4 | 5 |
| The cost of the medicine makes me less willing to dispose of it | 1 | 2 | 3 | 4 | 5 |
| I have a safe place to store medicines | 1 | 2 | 3 | 4 | 5 |

1. What you think about the following statements? For each statement, please select the option you consider most appropriate.

|  | Completely agree | Somewhat agree | Somewhat disagree | Completely disagree | I don’t know |
| --- | --- | --- | --- | --- | --- |
| My family/close ones think I should dispose of medicines properly | 1 | 2 | 3 | 4 | 5 |
| In my residential area, like others I am expected to dispose of medicines properly | 1 | 2 | 3 | 4 | 5 |
| Taking medicines out of the home makes my house safer (for myself, family members, and pets) | 1 | 2 | 3 | 4 | 5 |
| I feel that it is my duty to dispose of medicines properly to protect the environment | 1 | 2 | 3 | 4 | 5 |
| It is entirely my own decision, whether or not I dispose of medicines properly | 1 | 2 | 3 | 4 | 5 |
| I feel (or would feel) guilty if I did not dispose of medicines properly | 1 | 2 | 3 | 4 | 5 |
| I find it respectful and important that people dispose of medicines properly | 1 | 2 | 3 | 4 | 5 |

Thank you for your answers!

**Appendix 2.** Table of the response distribution of Likert-scale statements regarding the proper disposal of unnecessary and expired medicines (n = 5004).

| **Statement**  1 = Completely agree, 2 = Somewhat agree, 3 = Somewhat disagree, 4 = Completely disagree, 5 = I don’t know | **1** | **2** | **3** | **4** | **5** |
| --- | --- | --- | --- | --- | --- |
| **Environmental attitude towards medicines** | | | | | |
| ATT1. Pharmaceutical residues in nature pose a risk to the environment | 4370 (87.3) | 549  (11) | 14  (0.3) | 10  (0.2) | 61  (1.2) |
| ATT2. I am worried about the impact on human health of pharmaceutical residues in the environment | 3461 (69.2) | 1281 (25.6) | 113 (2.3) | 15  (0.3) | 134 (2.7) |
| ATT3. I am worried about the potential environmental impact of pharmaceutical products | 3638 (72.7) | 1146 (22.9) | 93  (1.9) | 15  (0.3) | 112 (2.2) |
| ATT4. The physician prescribing a medicine should consider the environmental impact of the product where possible | 1425 (28.5) | 2012 (40.2) | 527 (10.5) | 180 (3.6) | 860 (17.2) |
| ATT5. Leaflets in medicine packs describe the correct way to dispose of the medicine | 2152 (43) | 1416 (28.3) | 411 (8.2) | 119 (2.4) | 906 (18.1) |
| ATT6. Finland should set an example for other countries in reducing the impact of pharmaceutical products | 2957 (59.1) | 1477 (29.5) | 189 (3.8) | 49  (1) | 332 (6.6) |
| **Subjective norm** | | | | | |
| SN1. My family/close ones think I should dispose of medicines properly | 3130 (62.5) | 615 (12.3) | 75  (1.5) | 108 (2.2) | 1076 (21.5) |
| SN2. In my residential area. like others I am expected to dispose of medicines properly | 2029 (40.5) | 717 (14.3) | 108 (2.2) | 107 (2.1) | 2043 (40.8) |
| SN3. Taking medicines out of the home makes my house safer (for myself, family members, and pets) | 3054 (61) | 1011 (20.2) | 312 (6.2) | 221 (4.4) | 406 (8.1) |
| **Personal norm** | | | | | |
| PN1. I feel that it is my duty to dispose of medicines properly to protect the environment | 4338 (86.7) | 514 (10.3) | 50  (1) | 21  (0.4) | 81  (1.6) |
| PN2. I feel (or would feel) guilty if I did not dispose of medicines properly | 3648 (72.9) | 925 (18.5) | 116 (2.3) | 116 (2.3) | 199  (4) |
| PN3. I find it respectful and important that people dispose of medicines properly | 4614 (92.2) | 336 (6.7) | 10  (0.2) | 9  (0.2) | 35  (0.7) |
| **Perceived behavioral control** | | | | | |
| PBC1. I know where I can take the medicines for proper disposal | 4793 (95.8) | 173 (3.5) | 21  (0.4) | 10  (0.2) | 7  (0.1) |
| PBC2. It is easy for me to take the medicines for proper disposal | 4336 (86.7) | 506 (10.1) | 106 (2.1) | 41  (0.8) | 15  (0.3) |
| PBC3. I dispose of unnecessary medicines properly, regardless of whether I receive money for it (e.g. a deposit) | 4754 (95) | 179 (3.6) | 24  (0.5) | 21  (0.4) | 26  (0.5) |
| PBC4. The proper disposal of medicines is simple | 4311 (86.2) | 557 (11.1) | 88  (1.8) | 27  (0.5) | 21  (0.4) |
| PBC5. It is entirely my own decision, whether or not I dispose of medicines properly | 1381 (27.6) | 477 (9.5) | 739 (14.8) | 2210 (44.2) | 197 (3.9) |
| **Intention** | | | | | |
| INT1. I am happy to dispose of medicines in accordance with the instructions | 4631 (92.5) | 312 (6.2) | 25  (0.5) | 10  (0.2) | 26  (0.5) |
| INT2. I intend to dispose of medicines properly in the future | 4739 (94.7) | 223 (4.5) | 14  (0.3) | 7  (0.1) | 21  (0.4) |
| INT3. I am happy to dispose of medicines properly, if I know why it is worth doing so | 4355 (87) | 391 (7.8) | 65  (1.3) | 92  (1.8) | 101  (2) |
| INT4. I regard myself as committed to disposing of medicines properly in the future | 4665 (93.2) | 281 (5.6) | 26  (0.5) | 9  (0.2) | 23  (0.5) |
| **Behavior** | | | | | |
| BEH1. So far, I have disposed of medicines properly (or I would have done so, had I had any medicines to dispose of) | 4545 (90.8) | 353 (7.1) | 44  (0.9) | 39  (0.8) | 23  (0.5) |
| BEH2. I have not usually disposed of medicines properly^a^ | 152  (3) | 62  (1.2) | 237 (4.7) | 4428 (88.5) | 125 (2.5) |
| BEH3. I have been trying to reduce the amount of medicines in my home | 1551 (31) | 2094 (41.8) | 634 (12.7) | 258 (5.2) | 467 (9.3) |
| **Situational factors** | | | | | |
| SF1. I do not have time to dispose of medicines in the proper way | 71  (1.4) | 111 (2.2) | 286 (5.7) | 4412 (88.2) | 124 (2.5) |
| SF2. Difficulty going to the pharmacy or at the collection point hinders my proper disposal of medicines | 231 (4.6) | 361 (7.2) | 373 (7.5) | 3849 (76.9) | 190 (3.8) |
| SF3. The fact that it is not mandatory hinders the proper disposal of medicines | 239 (4.8) | 460 (9.2) | 294 (5.9) | 3709 (74.1) | 302  (6) |
| SF4. The cost of the medicine makes me less willing to dispose of it | 134 (2.7) | 338 (6.8) | 413 (8.3) | 3843 (76.8) | 276 (5.5) |
| SF5. I have a safe place to store medicines^a^ | 3107 (62.1) | 1476 (29.5) | 215 (4.3) | 112 (2.2) | 94  (1.9) |

^a^ In final analysis indicator was reverse coded

**Appendix 3.** Table of the measurement and structural model fit indices, and R^2^ values of intention and behavior.

| **Fit index** | **Cutoff value**^a^ | **The measurement model values  (standard deviation)** | **The structural model values  (standard deviation)** |
| --- | --- | --- | --- |
| RMSEA | < 0.06 | 0.039 (0.000) | 0.040 (0.000) |
| CFI | ≥ 0.95 | 0.971 (0.001) | 0.971 (0.001) |
| TLI | ≥ 0.96 | 0.966 (0.001) | 0.966 (0.001) |
| WRMR | < 0.9 | 1.990 (0.021) | 2.017 (0.021) |
| χ² | - | 2023.092  (35.399) | 2061.202  (34.371) |
| df | - | 231 | 234 |
| Χ^2^/df | ≤ 3 | 8.758 | 8.809 |
| **Variable** | **Estimate (R^2^)** | **Z-score** | **p-value** |
| INT | 0.850 | 52.903 | < 0.001 |
| BEH | 0.851 | 54.780 | < 0.001 |

^a^ Reference: Schreiber J, Nora A, Stage F, Barlow E, King J. Reporting Structural Equation Modeling and Confirmatory Factor Analysis Results: A Review, J Educ Res. 2006;99:323-338. doi:10.3200/JOER.99.6.323-338

BEH = Behavior, CFI = Comparative Fit Index, df = degrees of freedom, INT = Intention, RMSEA = Root Mean Square Error of Approximation, TLI = Tucker-Lewis Index, WRMR = Weighted Root Mean Square Residual, χ² = Chi-Square test

**Appendix 4.** Effect analysis of households’ proper disposal intention and behavior of unnecessary and expired medicines. Values are presented as standardized (StdY) path coefficients.

| **Hypo-thesis^a^** | **Path** | **Estimates** | **Standard error** | **Z-statistics** | **p-value** | **Result** |
| --- | --- | --- | --- | --- | --- | --- |
| H1 | ATT -> INT | 0.029 | 0.036 | 0.812 | 0.417 | Not supported |
| H2 | PN -> INT | 0.420 | 0.056 | 7.519 | < 0.001 | Supported |
| H3 | SN -> INT | 0.090 | 0.045 | 1.997 | 0.046 | Supported |
| H4-a | PBC -> INT | 0.351 | 0.044 | 7.930 | < 0.001 | Supported |
| H4-b | PBC -> BEH | 0.107 | 0.046 | 2.341 | 0.019 | Supported |
| H5-a | SF -> INT | -0.189 | 0.041 | -4.592 | < 0.001 | Supported |
| H5-b | SF -> BEH | -0.261 | 0.035 | -7.506 | < 0.001 | Supported |
| H6 | INT -> BEH | 0.617 | 0.036 | 17.373 | < 0.001 | Supported |

ATT = Environmental attitude towards medicines, BEH = Behavior, INT = Intention, PBC = Perceived behavioral control, PN = Personal norms, SF = Situational factors, SN = Subjective norms.

^a^ Hypotheses are presented in the article in Chapter 2.
